# Internet videos and colorectal cancer in mainland China: a content analysis

**DOI:** 10.1186/s12911-018-0711-x

**Published:** 2018-12-04

**Authors:** Shun Zhang, Yao Yang, Dongyi Yan, Biao Yuan, Xiaohua Jiang, Chun Song

**Affiliations:** Department of Gastrointestinal Surgery, Shanghai East Hospital (East Hospital Affiliated to Tongji University), 150 Jimo Road, Shanghai, 200120 People’s Republic of China

**Keywords:** Colorectal cancer, Internet, Youku, Mainland China

## Abstract

**Background:**

Colorectal cancer incidence and mortality have been increasing in China and as one of the most important health problems facing the nation. Adequate dissemination of correct information about colorectal cancer could help in reducing cancer-related morbidity and mortality. This study aims to assess the completeness and reliability of colorectal cancer-related information available on the video website of Youku in mainland China.

**Methods:**

Youku (https://www.youku.com/) was searched on September 15, 2016 for the search terms colorectal cancer. Only Chinese videos were included. Two reviewers independently evaluate the videos for characteristics, information source and usefulness. Content was analysed under six categories (aetiology, anatomy, symptoms, preventions, treatments and prognosis). Completeness was evaluated with a checklist developed by the researchers. Any discrepancies were resolved by consensuses. SPSS software was used to analyze data.

**Results:**

There were 242 videos with relevant information about colorectal cancer. The type of source were as follows: independent users, 118 (49%); health information web sites, 60 (25%); medical doctors, 31 (13%); news network, 22 (9%); and hospital/university, 11 (4%). In all, 57% of videos had useful information about colorectal cancer, 21% were misleading. Videos posted by medical doctors (*P* = 0.021) and health information web sites (*p* = 0.039) were less incomplete than videos by independent users. Of the Traditional Chinese medicine (TCM) videos, 97 (76%) had information about treatments of colorectal cancer. 30% TCM videos contain misleading information, whose misleading rate was higher than total’s (21%).

**Conclusions:**

The colorectal cancer videos in mainland China represented by Youku varied base on ownership and content and information incompleteness were fairly high. It is necessary that professionals adapt to the advanced technology and think useful methods to solve the variable quality of information of internet video websites in mainland China.

## Background

Cancer incidence and mortality have been increasing in China and have created a signifıcant number of health concerns [1]. Colorectal cancer ranks the fifth most commonly diagnosed cancer among male and female in China [2]. The ratio of estimated new colorectal cancer mortality incidence is 50.8% in China for 2015 [2] compared with 36.3% in the United States for 2016 [3]. This considerably higher ratio means cancer prevention and control in China lags behind some Western countries.

Up to 31 December 2016, it was reported that 731 million Chinese internet users, and more than 695 million people were using mobile devices to quick browse online information. Over 570 million online video users accounted for three-quarters of total internet users [4]. Health and medical treatment has been the most popular science topics in mainland China [5]. Freely available video websites, such as YouTube, are popular sources of information dissemination with more than 100 million viewers every day [6]; however, YouTube is blocked in China because of Chinese internet censorship.

Chinese people prefer online video websites, such as Yoku, iQiyi, Sohu Tv or Tencent Video. Youku is the most popular source of video blogs and short original videos uploaded by individuals in mainland China [7]. Youku initially emphasized user-generated content. The average number of daily video views was 1.18 billion [8]. The number of monthly active users was over 500 million, and 60% of audiences were male [7]. Youku features the same kinds of videos on YouTube and is considered the largest Chinese video broadcast site. Similar to YouTube, the posted videos are not peer control, could be uploaded from different sources and are likely to be of variable quality [9].

Many studies reported that video broadcast sites have positive and negative effects on health information dissemination. Some videos can provide useful resources for knowledge and were used by medical students as a learning resource [10, 11]. Videos may promote misleading information, such as disparaging vaccinations [9] and describing ineffective or potentially dangerous natural therapies for gallstone disease [12]. Not only were audience attempting therapies that may be harmful, but they were not going in for accurate therapy, which can lead to other complications.

The use of video broadcasting sites as a source of information in disease areas, especially in colorectal cancer in mainland China, has not been evaluated. Thus, the present study aimed at evaluating the completeness and reliability of Chinese-language colorectal cancer-related information available on the video website of Youku in mainland China; assess the overall quality of online information on colorectal cancer; and share our thoughts on important future directions for managing information about colorectal cancer on websites of mainland China.

## Method

We searched Youku (www.youku.com) on November 15, 2016 to locate video clips containing relevant information about colorectal cancer in human patients. The keyword “colorectal cancer” was used to identify related video clips. Videos that were duplicated, not in Chinese and not directly related to the investigated condition were excluded.

We included all unique videos with Chinese language content that contained any message about human colorectal cancer. All videos were downloaded and saved. We assessed each video according to the following characteristics: duration, ownership, number of views, video quality, and colorectal cancer content. Ownership was classified by medical doctor, hospital/university, news network, health information website or independent user.

All videos were viewed and analysed for content by 2 reviewers, and disagreements were resolved by an arbitrator. All researchers had medical background and specialized in gastrointestinal surgery. All researchers had finished their respective residencies at general hospital and had enough experience in the diagnosis and management of colorectal cancer. The reviewers classified the videos as useful, misleading or useless, as defined by the following: **useful**—containing scientifically correct information about any aspect of the disease: symptoms, treatment, and prevention; **misleading—** containing scientifıcally unproven information; **useless**—without containing the any aspect of colorectal cancer or addressing personal experience. If the video included trustable and misleading information at the same time, the videos were categorized as “misleading”.

We assessed the quality of each video using a completeness score (Table 1). Two reviewers viewed each video in all content areas (aetiology, anatomy, symptoms, preventions, treatments and prognosis). At present, no validated tool for this purpose exists in the literature. Any disagreements were resolved with consensuses.

**Table 1:** Completeness checklist

Content	Description
Aetiology	Precancerous lesion
	Heredity
	Eating habits
Anatomy	–
Symptoms	Stool change
	Altered bowel habits
	Abdominal pain
	Abdominal mass
	Systemic symptoms
Preventions	Screening
	Daily habits
Treatments	Surgery
	Chemotherapy
	Radiotherapy
	Traditional Chinese medicine
Prognosis	TNM stage
	Perioperative treatments
	Others

Traditional Chinese medicine (TCM) has been is deeply embedded in the populations of China and applied to the prevention and treatment of various diseases from ancient times until now. TCM is promoted and institutionalized by the Chinese government, has spread to more than 100 countries and has grown into an international industry [13]. For this reason, we also analysed TCM content regarding colorectal cancer in our study. Inter-observer agreement was evaluated with a kappa coefficient. Differences between groups were compared with a one-way ANOVA. Data analysis was performed with SPSS Version 16 Software. If the *p*-value is less than 0.05, the result was considered to be significant.

## Result

A Youku search revealed 348 videos for colorectal cancer. Videos were removed for a variety of reasons (Table 2). Video duplication and not being in Chinese were the two main reasons. Of the 348 videos screened, 242 videos met the inclusion criteria.

**Table 2:** Reasons for excluding videos

Reason for exclusion	No.
No audio	1
No video	2
Not in Chinese	15
Not related to subject	3
Duplicate	85
Total exclusions	106

### Ownership

A total of 49% of the videos were posted on the website by independent users. Health information websites were responsible for uploading 25% of the total videos. The videos contributed by medical doctors were only 13% but higher than other owner videos by max viewership and mean viewership (Table 3). This difference among groups was statistically significant (*p* < 0.05).

**Table 3:** Sources and classification of detected videos

Source	Total videos	Max viewership	Min viewership	Mean viewership
Independent user	118	53,455	8	10,621
Health information web site	60	60,234	167	15,390
Medical doctor	31	61,132	12,583	23,893
Hospital/University	11	20,343	670	8783
News network	22	57,890	4791	9321

### Information reliability

The 242 included videos were classified as useful (136 [57%]), misleading (51 [21%]), and useless (55 [22%]) according to medical content (Table 4). The kappa coefficient statistics of agreement of these videos was 0.88.

**Table 4:** Sources and classification of detected videos

Ownership	Total Videos	Useful (Mv)	Misleading (Mv)	Useless (Mv)
Independent users	118	43 (7689)	41 (18123)	34 (5283)
Health information web site	60	48 (17517)	4 (895)	8 (9876)
Medical doctors	31	28 (24588)	1 (12583)	2 (19812)
Hospital/University	11	11 (8783)	0 (−)	0 (−)
News network	22	6 (7302)	6 (4567)	11 (12583)
Mean duration (min ± SD)	4.0 ± 2.3	5.5 ± 3.7	4.3 ± 2.1	3.7 ± 3.1

Abbreviation: *SD*, standard deviation; Mean viewership: *Mv*

The number of videos containing misleading information was 51. A large part (41 [80%]) were amateur videos about personal experiences and emotions. The mean duration of the videos was 4.0 min with no significant differences between useful and misleading videos or between useful and irrelevant videos (*p* < 0.05).

### Content

Useful videos were analysed based on the information they contained. In all of the categories, treatments were the most frequently covered topic (70%), followed in descending order by symptoms (33%), prognosis (26%), anatomy (20%), preventions (15%) and aetiology (11%). Table 5 shows the information completeness scores. Videos by medical doctors (*p* = 0.021) and health information websites (*p* = 0.039) sources were significantly more complete than those posted by independent users.

**Table 5:** Completeness score

Completeness score	No	Mean ± SD
Aetiology	15	1.53 ± 0.51
Anatomy	27	–
Symptoms	43	2.77 ± 1.23
preventions	21	1.24 ± 0.44
Treatments	95	2.07 ± 0.83
Prognosis	35	1.77 ± 0.69
Total (max = 17)	136	3.07 ± 1.94

### Traditional Chinese medicine

There were 128 videos containing TCM from diagnosis to treatment. Of the TCM videos, 97 (76%) had information about treatments of colorectal cancer. Among these videos, 10 included TCM and Western medicine at the same time. The information reliability is shown in Table 6. Medical doctors and university provided more reliable information than others (*p* < 0.05). A total of 30% TCM videos contain misleading information, and this misleading rate was higher than the totals (21%). Among the videos containing both TCM and Western medicine, the misleading rate was as high as 90%. Most of the videos exaggerated the actual effect of TCM and understated therapies, except for the health information websites mean viewership (798: 895). The other sources’ mean viewership in TCM were higher than those containing both TCM and non-TCM videos.

**Table 6:** Treatments of Traditional Chinese medicine

	Total videos	Useful (Mv)	Misleading (Mv)	Useless (Mv)
Independent users	62	23 (13532)	35 (20021)	4 (6577)
Health information web site	24	16 (23122)	3 (798)	5 (1201)
Medical doctors	7	7 (27349)	0 (−)	0 (−)
Hospital/University	1	1 (13653)	0 (−)	0 (−)
News network	3	1 (12021)	0 (−)	2 (13216)

Abbreviation: Mean viewership: *Mv*

## Discussion

Colorectal cancer ranks as the fifth leading cause of cancer death among both male and female in mainland China. Because the population of China accounts for one fifth of the global world, colorectal cancer cases in China account for 22% of all newly diagnosed cases and 27% of all deaths from worldwide [14]. The effectiveness of prevention, early detection, and management of colorectal cancer is not only important for China but also for the world.

Internet video websites can provide useful diagnostic, treatment and preventative medical services information. Previous research has evaluated YouTube as an important source of information on disease topics [15]. Although YouTube is blocked due to many reasons in mainland China, there are many similar internet video websites delivering the same functionality, such as Youku. To the best of our knowledge, no study has been performed to assess the accuracy and usefulness of internet videos as a source of healthy information for colorectal cancer in mainland China.

In this study, we selected Youku.com as the target video website, which is ranked the largest Chinese video broadcast site. The website of Youku not only focuses on professionally produced videos but also emphasizes user-generated content. The monthly unique visitors of Youku were 2,6376,000,000 according to the data of October in 2016 [7].

Our study demonstrates approximately 242 videos addressing colorectal cancer were provided by different sources. Independents users represent the greatest number of sources. The content was mainly about personal experiences in surgical procedures or hospital stays. Our results also show that Chinese medical doctors and health related institutions comprising 17% of colorectal cancer videos do not pay sufficient attention to the platform for the distribution of information. Doctors in china frequently experience work overload, tend to work overtime and experience energy deficiencies, which seem to be one of reasons for this phenomenon [16]. The videos that were viewed most often were the videos posted by doctors followed by health information websites. This indicates that people are more interested in a professionals experience regarding disease rather than their peers.

As the content of most videos often lacks peer or institutional quality review, many may not be subject to quality controls and may not be evidence-based; thus, it is not surprising that a majority of this content is misleading or irrelevant. According to previous studies, the dissemination of inaccurate information by video websites differs from diseases. A total of 56.5% of the video information on cholecystolithiasis [11], 16.2% on H1N1 influenza [17] and 1.6% on acute appendicitis in children [18] on YouTube were misleading. In our study, it was demonstrated that only one-fifth of website videos contain no scientifically oriented information. Only 36% of the independent users videos reviewed were considered to be useful compared with 90% useful doctors’ videos.

The most commonly watched videos from independent users were those that contained misleading information, while the lowest number of views were from medical doctors and health information websites. These results also indicated that effective regulatory measures are needed to control scientifically accredited information. If misleading videos were less viewed by audiences, the harm might be reduced.

Regarding videos addressing colorectal cancer, it is highly difficult for laypeople or patients to distinguish between useful videos or those containing no accurate information. Our result indicates that an important element to assess the reliability of videos regarding colorectal cancer may be the ownership. If academic institutions represent the source, such as hospital/university or medical doctors, the videos may be regarded to be trustworthy on the basis of content [19]. The result is similar to those of other studies conducted outside of mainland China [15, 16].

We found that the average completeness scores were only 18% with a combination of aetiology, anatomy, symptoms, preventions, treatments and prognosis. Most of the included website videos only contained one of the above-mentioned categories. In all of the categories, treatments were the most frequently covered topic (70%). It is unlikely to expect all videos to comprehensively cover all aspects of colorectal cancer; therefore, it should be deemed that some videos, whilst incomplete, do contain precise and valuable content. Our results indicated that videos from medical doctors and health related institutions have significantly higher completeness scores than those posted by independent users. This result may suggest that videos posted by layperson mainly aim a more social goal and videos posted by health and medical organizations commonly take a more educational purpose. The study indicated that professionals should utilize their expertise and contribute to more high-quality videos for patients as information sources in mainland China.

When video contents were analyzed, the most universal topic were the treatment aspects of the colorectal cancer. This finding may indicate that most publishers thought that treatment factors are the most important component of colorectal cancer. Surgery, chemotherapy and radiotherapy have been the mainstay of colorectal cancer treatment. Approximately 70% of videos contained one of the above subjects. As the country of origin and application of TCM, China has a unique TCM theoretical system and effective treatment methods. In mainland China, TCM has been recognized as additional treatment methods for colorectal cancer [20]. Our study shows that approximately 128 videos were about the anticancer properties of traditional Chinese medicine.

In oncology, TCM is believed to have great healing properties such as exerting specific anticancer activity or chemosensitisation to help in the individualization of anticancer treatment [21, 22]. Chinese cancer patients frequently believe that herbs of TCM can help them against suffering from complications and to live well. Doctors trained in Western medicine published fewer videos than doctors trained in Chinese medicine. However, 30% of TCM videos contained misleading information that exaggerated actual effects and propaganda error messages, such as curing colorectal cancer. The highest total and misleading number of videos were posted by independent users. The meanest viewership was also from independent users. The misleading rate was higher than total misleading rate (21%). There have been a large number of controlled clinical studies published in Chinese literature, but high-level evidence for the clinical efficacy of TCM is still lacking [23]. Mistakes were often found in professorial papers and in internet videos.

Colorectal cancer is characterized by high prevalence, a long asymptomatic period and eminently treatable precancerous lesions which, taken together, suggests that screening is a prudent option in mainland China [24]. For this reason, facilitating the earlier diagnosis of colorectal cancer may have a more immediate impact on the existing cancer burden in mainland China. A total of 21 videos contained colorectal cancer screening, which represented only 15% of all useful videos. Almost all screening videos address the importance of a Faecal Occult Blood Test, digital rectal exam, and colorectaloscopy.

Despite the rising colorectal cancer incidence, public awareness is still low in mainland China. Chinese internet websites, such as Youku, provide a different medium to disseminate colorectal cancer information to the public by video instead of written text. The written healthy information is commonly at a considerably higher reading level for Chinese patients. This video-based information source can help them and their caregivers get better understanding. Use of the internet for colorectal cancer information is likely to increase. It is necessary that professional individuals and academic institutions adapt to the advanced technology and think useful methods to solve the variable quality of information uploaded on internet video websites in mainland China. To maximize the potential of video-based information and minimize the quantity misleading or unhelpful information, multilateral efforts between doctors, governments and websites are needed.

### Limitations

First, the main bias of our study was the subjectivity of judgement. There were no validated tools for assessing video data. Therefore, our classification method was subjective. However, the kappa statistic indicated quite high agreement between two reviewers. Second, there was no website, such as YouTube, with a clearly dominant position in China. Selecting only one Chinese video website’s data may lead to some bias. Youku was the most popular website and had the largest audience in China. Youku in mainland China may still reflect the reliability of information available on video websites. Third, our results comprise a snapshot of information distribution to illustrate the quality of internet video at one point in time in China mainland, and these results may change according to the videos that can be added or removed with time.

## Conclusions

Colorectal cancer videos represented by Youku in mainland China varied significantly by ownership and content and information incompleteness were fairly high. It is necessary that professionals adapt to the advanced technology and think useful methods to solve the variable quality of information uploaded on internet video websites in mainland China.

## Abbreviation

TCM: Traditional Chinese medicine
